# Analysis of the disease burden of vertebral fractures in China and worldwide from 1990 to 2021 and trend forecast to 2035

**DOI:** 10.1186/s41043-025-01114-8

**Published:** 2025-10-28

**Authors:** Hongwen Gu, Kangen Han, Junchao Li, Zhihao Zhang, Le Xing, Yin Hu, Yanchun Xie, Hailong Yu

**Affiliations:** 1 Department of Orthopedics, General Hospital of Northern Theater Command of Chinese PLA, Shenyang, 110016 Liaoning China; 2 Graduate School of Dalian Medical University, Dalian, 116051 Liaoning China

**Keywords:** Vertebral fractures, Trend, Incidence, Prevalence, Years lived with disability

## Abstract

**Methods:**

Using the Global Burden of Disease (GBD) 2021 database, we analyzed the epidemiological trends of VFs in China from 1990 to 2021 and further explored the disease burden characteristics by age and sex. The Joinpoint regression model was employed to calculate the average annual percentage change (AAPC) to analyze the trends. The Bayesian Age-Period-Cohort (BAPC) model was used to predict the changes in standardized rates from 2022 to 2035. Additionally, decomposition analysis was conducted to investigate the impacts of aging, population growth, and epidemiological changes on the disease burden.

**Results:**

In 2021, the number of incident cases, prevalent cases, and YLDs of VFs globally were 7,497,446, 5,371,438, and 545,923, respectively, which increased by 28.03%, 57.96%, and 54.67% compared with 1990. From 1990 to 2021, the global ASIR, ASPR, and ASYR all decreased (AAPC= -0.72%, -0.73%, and − 0.75%, respectively, *P* < 0.05). The BAPC model also predicted a similar downward trend from 2022 to 2035. In 2021, the number of incident cases, prevalent cases, and YLDs of VFs in China were 1,194,465, 717,078, and 74,079, respectively, which increased by 52.28%, 113.66%, and 107.21% compared with 1990. From 1990 to 2021, the ASIR in China showed a non-significant increase (AAPC = 0.45%, *P* > 0.05), while the ASPR and ASYR both increased significantly (AAPC = 0.53% and 0.51%, respectively, *P* < 0.05). The BAPC model predicted a downward trend in ASIR, ASPR, and ASYR from 2022 to 2035 Gender analysis revealed that the disease burden is more prominent among middle-aged men and elderly women. The age-group analysis showed that the peak age groups for the number of incident cases, prevalent cases, and YLDs all increased, and CIR, CPR, and CYR all increased with age. The decomposition analysis revealed that population growth was the primary factor driving the increase in the number of VFs cases globally, while population growth and aging were the main factors contributing to the increase in the number of VFs cases in China.

**Conclusion:**

The disease burden of vertebral fractures is significant and cannot be ignored. China should develop targeted public health strategies based on its demographic structure and socio-economic development, focusing on the prevention and treatment of vertebral fractures in middle-aged men and elderly women to achieve the goal of universal health.

**Supplementary Information:**

The online version contains supplementary material available at 10.1186/s41043-025-01114-8.

## Introduction

Vertebral fractures (VFs) are defined as the occurrence of breaks or ruptures in the vertebrae that constitute the spine. The spine consists of the cervical, thoracic, lumbar, sacral, and coccygeal vertebrae, and fractures can occur in any part of these areas [1]. ​Common causes of VFs include high-energy trauma (such as motor vehicle accidents, falls from a height, sports injuries) and conditions that lead to bone fragility, such as osteoporosis [2, 3]. Based on the mechanism and morphology of the fracture, VFs can be classified into the following types: Compression fractures, Burst fractures, and Chance (flexion/distraction) fractures [4, 5].

With the global population growth and aging, the disease burden of VFs is becoming more serious. In 2019, the global incidence of vertebral fractures was 8.6 million, representing a 38% increase from 1990 [6]. In the United States alone, the total surgical cost for treating VFs reached 1.06 billion US dollars in 2014 [7]. From 2017 to 2020, 5.2 million Americans aged 50 and above experienced VFs [8]. In China, the incidence rate of VFs among urban residents aged 50 and above increased by 1.79 times from 2013 to 2017, and the medical costs for treating VFs soared from 92.74 million US dollars in 2013 to 505 million US dollars in 2017 [9]. Model predictions indicate that by 2050, China will see an additional 3.01 million cases of VFs, with related medical system costs exceeding 20 billion US dollars [10]. Moreover, research has shown that the mortality rate among patients with VFs is significantly higher than that of the general population [11].

China, with its large population, offers a distinctive and essential background for comprehending the evolving burden of VFs. Although several studies have investigated the burden of VFs in China [12–14], there is a notable lack of comprehensive and long-term analyses to assess its temporal trends. This deficiency underscores the necessity for a more nuanced understanding of the burden of VFs and its developmental trajectory in China to guide public health policies and resource allocation. In order to bridge this gap, this study employed GBD 2021 data to perform a detailed analysis of the burden of VFs in China from 1990 to 2021 and compared it with global trends. The results are intended to offer policymakers key insights to facilitate evidence-based planning of preventive strategies and effective allocation of public health resources.

## Methods

### Data source

The data for this study were derived from the Global Burden of Disease Study 2021 (GBD 2021) (https://ghdx.healthdata.org/gbd-2021), which provides data on 371 diseases and injuries from 100,983 data sources, as well as the corresponding 88 major risk factors, with VFs being one of the injuries. The authors extracted data on the burden of VFs in China and globally from 1990 to 2021, including data by sex and age (20 age groups). The main indicators included: number of incident cases, number of prevalent cases, Years Lived with Disability (YLDs), crude incidence rate (CIR), crude prevalence rate (CPR), crude YLDs rate (CYR), age-standardized incidence rate (ASIR), age-standardized prevalence rate (ASPR), and age-standardized YLDs rate (ASYR). The database used the DisMod-MR 2.1 statistical modeling tool to standardize global population and epidemiological data and employed a 95% uncertainty interval (UI) to ensure the scientific validity and consistency of the estimates.

### Statistical analysis

The Joinpoint regression analysis was employed to compute the annual percentage change (APC) and the average annual percentage change (AAPC) for the period 1990–2021, as well as their respective 95% confidence intervals (CI). If the AAPC estimate and its lower CI limit are both greater than 0, it indicates a significant upward trend; if the AAPC estimate and its upper CI limit are both less than 0, it indicates a significant downward trend; if neither of the above conditions is met, it is considered that the age-standardized rate does not change significantly over time. The Bayesian age-period-cohort model (BAPC) was used to predict the ASIR, ASPR, and ASYR of VFs from 2022 to 2035. The standard population data and estimated data used for the prediction analysis were derived from the data files in the GBD database [15, 16]. Decomposition analysis was applied to further assess the contributions of various factors to the burden of Fracture of vertebral column, dividing the disease burden into three influencing factors: aging, population growth, and epidemiological changes.

## Results

### The burden of VFs in China and globally

#### Trends in the incidence burden of VFs in China and globally

The number of incident cases of VFs globally was 5,856,226 (95% UI: 4,615,149-7,402,697) in 1990 and 7,497,446 (95% UI: 5,834,963-9,737,255) in 2021, representing an increase of 28.03%. The ASIR of global VFs was 115.75 (95% UI: 91.28–145.92.28.92) in 1990 and 92.75 (95% UI: 72.12–119.99.12.99) in 2021. From 1990 to 2021, the ASIR of global VFs showed a downward trend, with an average annual decrease of 0.72% (95% CI: −0.89% to −0.56%), and the difference was statistically significant (*P* < 0.05). The number of incident cases of VFs in China was 784,386 (95% UI: 598,122-1,027,222) in 1990 and 1,194,465 (95% UI: 888,994-1,594,120) in 2021, representing an increase of 52.28%. The ASIR of VFs in China was 68.32 (95% UI: 52.49–88.79) in 1990 and 76.38 (95% UI: 57.33–102.58.33.58) in 2021. From 1990 to 2021, the ASIR of VFs in China showed no significant trend, with an average annual increase of 0.45% (95% CI: −0.29% to 1.20%), and the difference was not statistically significant (*P* > 0.05). From 1990 to 2021, the incidence burden of VFs in China increased at a higher rate compared to the global level. While the global ASIR showed a significant decline, the ASIR in China increased slightly each year (Table 1).

#### Trends in the prevalence burden of VFs in China and globally

The number of prevalent cases of VFs globally was 3,400,460 (95% UI: 2,958,279-3,925,843) in 1990 and 5,371,438 (95% UI: 4,703,837-6,196,132) in 2021, representing an increase of 57.96%. The ASPR of global VFs was 81.55 (95% UI: 71.55–93.04) in 1990 and 65.19 (95% UI: 56.89–75.28) in 2021. From 1990 to 2021, the ASPR of global VFs showed a downward trend, with an average annual decrease of 0.73% (95% CI: −0.83% to −0.63%), and the difference was statistically significant (*P* < 0.05). The number of prevalent cases of VFs in China was 335,614 (95% UI: 277,475 − 397,432) in 1990 and 717,078 (95% UI: 619,114–835,347) in 2021, representing an increase of 113.66%. The ASPR of VFs in China was 34.89 (95% UI: 29.81–40.53) in 1990 and 40.51 (95% UI: 34.42–47.56) in 2021. From 1990 to 2021, the ASPR of VFs in China showed an upward trend, with an average annual increase of 0.53% (95% CI: 0.24% to 0.83%), and the difference was statistically significant (*P* < 0.05). From 1990 to 2021, the prevalence burden of VFs in China increased at a higher rate compared to the global level. While the global ASPR showed a significant decline, the ASPR in China increased significantly (Table 1).

#### Trends in the YLDs burden of VFs in China and globally

The number of YLDs for global VFs was 352,960 (95% UI: 235,711 − 491,606) in 1990 and 545,923 (95% UI: 366,571–757,099) in 2021, representing an increase of 54.67%. The ASYR for global VFs was 8.33 (95% UI: 5.61–11.49) in 1990 and 6.62 (95% UI: 4.43–9.2) in 2021. From 1990 to 2021, the ASYR for global VFs showed a downward trend, with an average annual decrease of 0.75% (95% CI: −0.85% to −0.65%), and the difference was statistically significant (*P* < 0.05). The number of YLDs for VFs in China was 35,750 (95% UI: 23,555 − 51,348) in 1990 and 74,079 (95% UI: 49,798 − 104,323) in 2021, representing an increase of 107.21%. The ASYR for VFs in China was 3.65 (95% UI: 2.42–5.12) in 1990 and 4.19 (95% UI: 2.78–5.96) in 2021. From 1990 to 2021, the ASYR for VFs in China showed an upward trend, with an average annual increase of 0.51% (95% CI: 0.21% to 0.81%), and the difference was statistically significant (*P* < 0.05). From 1990 to 2021, the YLDs burden of VFs in China increased at a higher rate compared to the global level. While the global ASYR showed a significant decline, the ASYR in China increased significantly (Table 1).

**Table 1:** Burden of VFs in China and globally in 1990 and 2021 and trends from 1990 to 2021

Location	Measure	1990		2021		1990-2021AAPC
		All-ages cases	Age- standardized rates per 100,000 people	All-ages cases	Age-standardized rates per 100,000 people	
		*n* (95% UI)	*n* (95% UI)	*n* (95% UI)	*n* (95% UI)	*n* (95% CI)
China	Incidence	784,386 (598122–1027222)	68.32 (52.49–88.79)	1,194,465(888994–1594120)	76.38 (57.33–102.58.33.58)	0.45 (−0.29–1.20)
	Prevalence	335,614 (277475–397432)	34.89 (29.81–40.53)	717,078 (619114–835347)	40.51 (34.42–47.56)	0.53 (0.24–0.83)*
	YLDs	35,750 (23555–51348)	3.65 (2.42–5.12)	74,079 (49798–104323)	4.19 (2.78–5.96)	0.51 (0.21–0.81)*
Global	Incidence	5,856,226(4615149–7402697)	115.75 (91.28–145.92.28.92)	7,497,446 (5834963–9737255)	92.75 (72.12–119.99.12.99)	−0.72 (−0.89 – −0.56)*
	Prevalence	3,400,460(2958279–3925843)	81.55 (71.55–93.04)	5,371,438 (4703837–6196132)	65.19 (56.89–75.28)	−0.73 (−0.83 – −0.63)*
	YLDs	352,960 (235711–491606)	8.33 (5.61–11.49)	545,923 (366571–757099)	6.62 (4.43–9.2)	−0.75 (−0.85 – −0.65)*

*Indicates *p*-values < 0.05, denoting statistically significant results

### Joinpoint regression analysis of the burden of VFs in China and globally

**Fig. 1:**
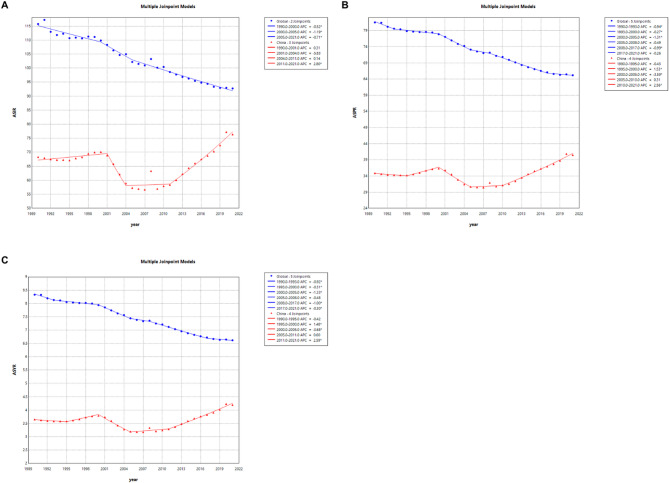
Joinpoint regression analysis of ASIR, ASPR, and ASYR of VFs in China and globally from 1990 to 2021. (**A**) ASIR; (**B**) ASPR; (**C**) ASYR. (* indicates *P* < 0.05, blue curves represent global trends, and red curves represent China)

Joinpoint regression analysis emphasized the significance of the trends in ASIR, ASPR, and ASYR of VFs in China and globally from 1990 to 2021. Globally, the ASIR, ASPR, and ASYR showed a downward trend from 1990 to 2021, with the most significant decline occurring between 2000 and 2005, with corresponding APC values of −1.19, −1.31, and − 1.33, all of which were statistically significant (*P* < 0.05). In China, the same declining trend was observed during the corresponding period, but it was more pronounced, with APC values of −5.38, −3.59, and − 3.68, all of which were statistically significant (*P* < 0.05). However, the overall trend for ASIR, ASPR, and ASYR in China was upward, with the most significant increase occurring between 2011 and 2021, with APC values of 2.8, 2.56, and 2.59, all of which were statistically significant (*P* < 0.05). By comparing these trends, Joinpoint regression highlighted the more pronounced variability and unique trajectory of China compared to the global pattern. This analysis is crucial for identifying periods of significant change, enabling policymakers to design targeted interventions to address these temporal and regional nuances (Fig. 1).

### Forecast for the burden of VFs in China and globally from 2022 to 2035

**Fig. 2:**
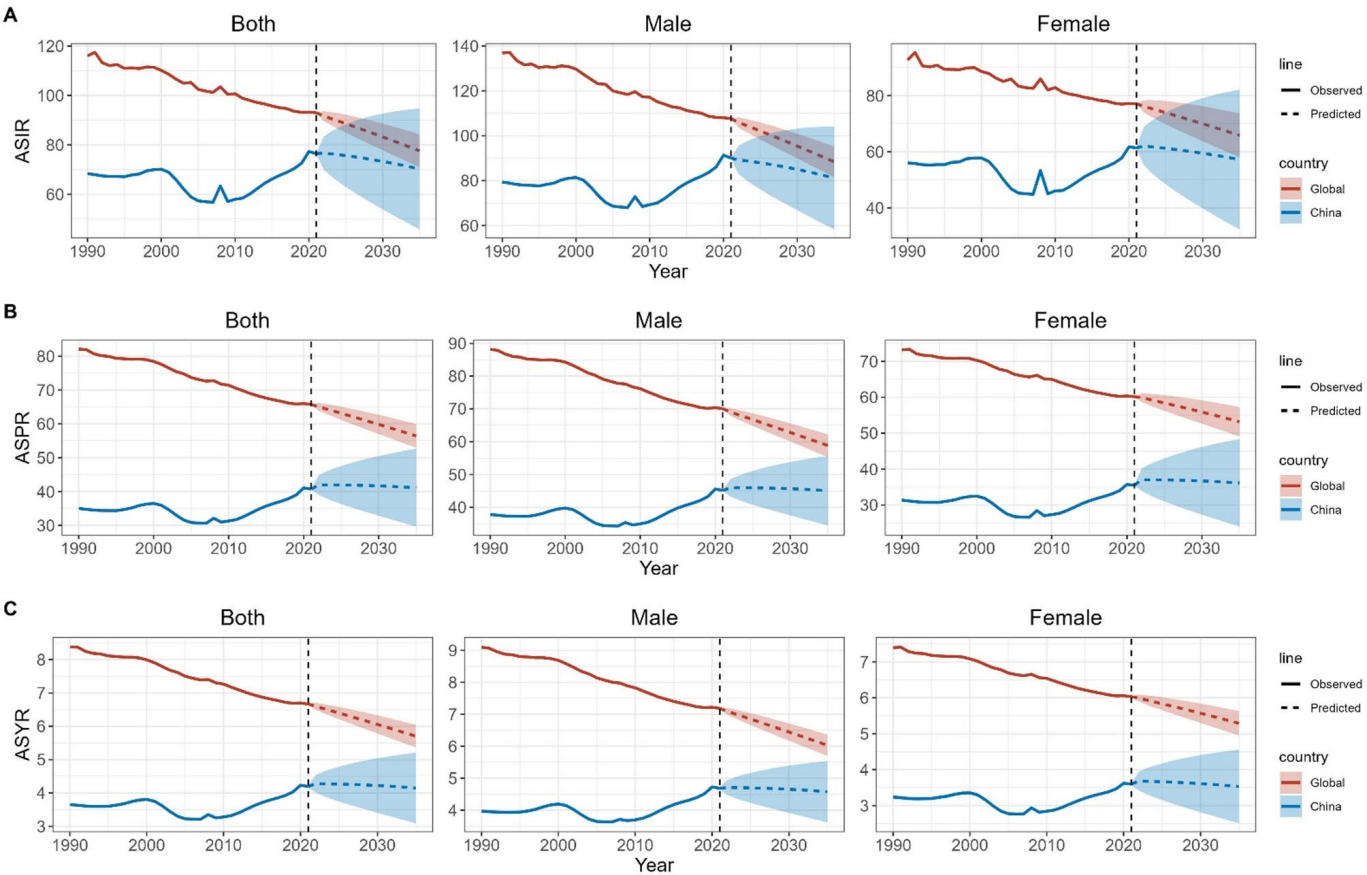
Forecast of ASIR, ASPR, and ASYR for VFs in China and globally from 2022 to 2035. (**A**) ASIR; (**B**) ASPR; (**C**) ASYR

It is projected that from 2022 to 2035, the ASIR, ASPR, and ASYR of VFs in China and globally will show a downward trend. By 2035, the ASIR for global VFs overall, in males, and in females will be 77.62 (95% UI: 70.91–84.33), 88.36 (95% UI: 81.54–95.18), and 65.82 (95% UI: 58–73.64.64), respectively. The ASPR will be 56.41 (95% UI: 52.89–59.93), 58.79 (95% UI: 55.38–62.19), and 53.15 (95% UI: 49.06–57.24), respectively. The ASYR will be 5.7 (95% UI: 5.37–6.04), 6.03 (95% UI: 5.7–6.36), and 5.29 (95% UI: 4.95–5.63), respectively. For China, the ASIR for VFs overall, in males, and in females will be 70.27 (95% UI: 45.81–94.73), 81.15 (95% UI: 58.27–104.03.27.03), and 57.22 (95% UI: 32.29–82.15), respectively. The ASPR will be 41.15 (95% UI: 29.66–52.64), 45.06 (95% UI: 34.45–55.68), and 36.16 (95% UI: 23.99–48.33), respectively. The ASYR will be 4.15 (95% UI: 3.09–5.21), 4.58 (95% UI: 3.62–5.53), and 3.53 (95% UI: 2.51–4.56), respectively. It can also be observed that, both globally and in China, the ASIR, ASPR, and ASYR in males are higher than those in females (Fig. 2).

### Analysis of VFs burden in China and globally by age group

**Fig. 3:**
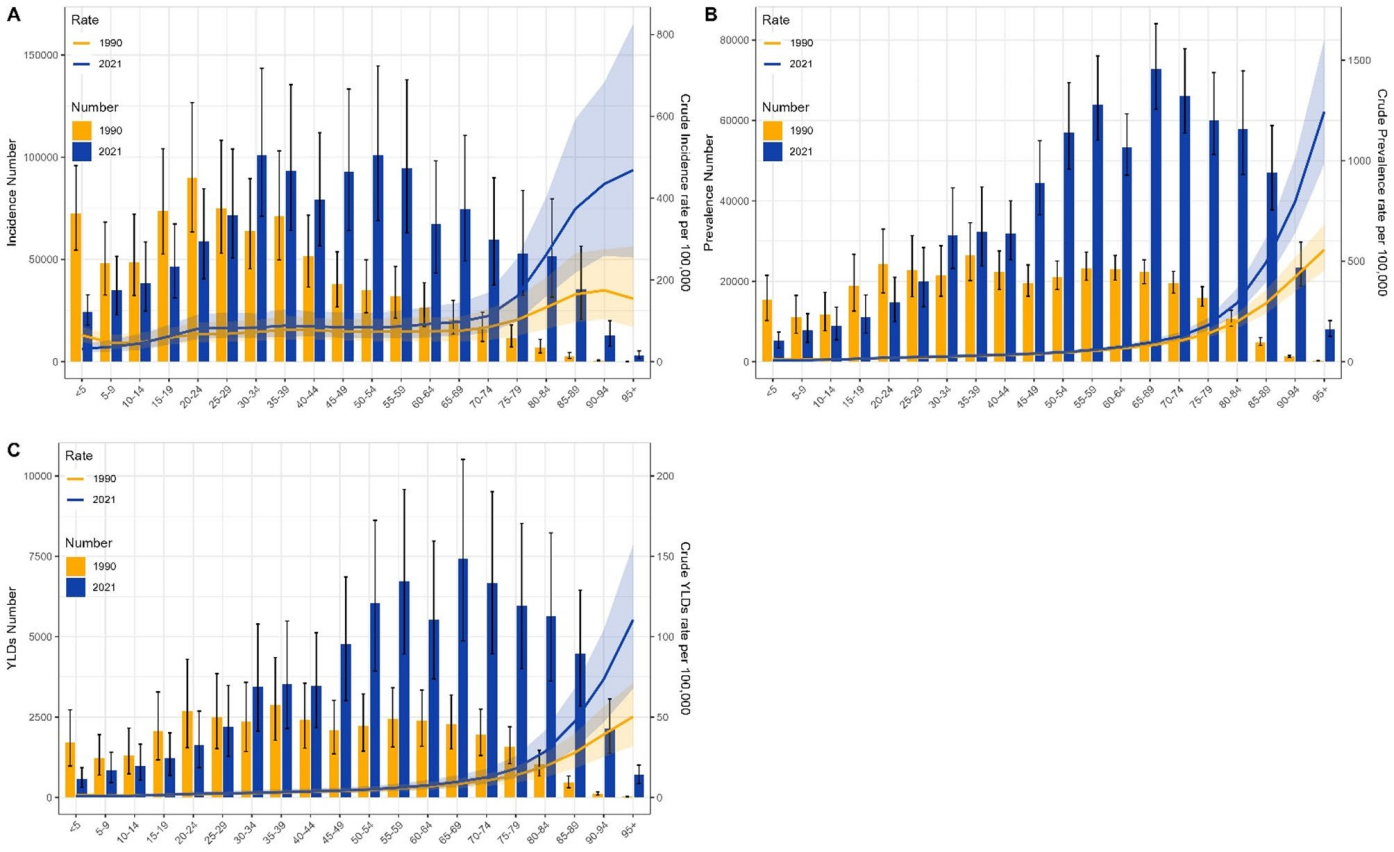
Burden of VFs in China by age group in 1990 and 2021. (**A**) Incidence number and CIR; (**B**) Prevalence number and CPR; (**C**) YLDs number and CYR

Globally, the age group with the highest incidence of VFs was 20–24 in both 1990 and 2021. The age group with the highest prevalence shifted from 75 to 79 in 1990 to 80–84 in 2021, and the age group with the highest YLDs shifted from 65 to 69 in 1990 to 70–74 in 2021. The peak age group for CIR of VFs was 90–94 in 1990 and 95 + in 2021, while the highest CPR and CYR were in the 95 + age group in both years. Globally, CIR, CPR, and CYR of VFs all increased with age (Supplementary Fig. 1) In China, the age group with the highest incidence of VFs shifted from 20 to 24 in 1990 to 50–54 in 2021. The age groups with the highest prevalence and YLDs both shifted from 35 to 39 in 1990 to 65–69 in 2021. The peak age group for CIR of VFs was 90–94 in 1990 and 95 + in 2021, while the highest CPR and CYR were in the 95 + age group in both years. In China, CIR, CPR, and CYR of VFs all increased with age, and in the elderly stage, CIR, CPR, and CYR in 2021 were significantly higher than in 1990 (Fig. 3)It can be observed that, both globally and in China, the peak age groups for incidence, prevalence, and YLDs have increased. In China, the span of the peak age groups is larger, and CIR, CPR, and CYR are positively correlated with age, indicating a significant burden of aging.

### Analysis of VFs burden in China and globally by gender

**Fig. 4:**
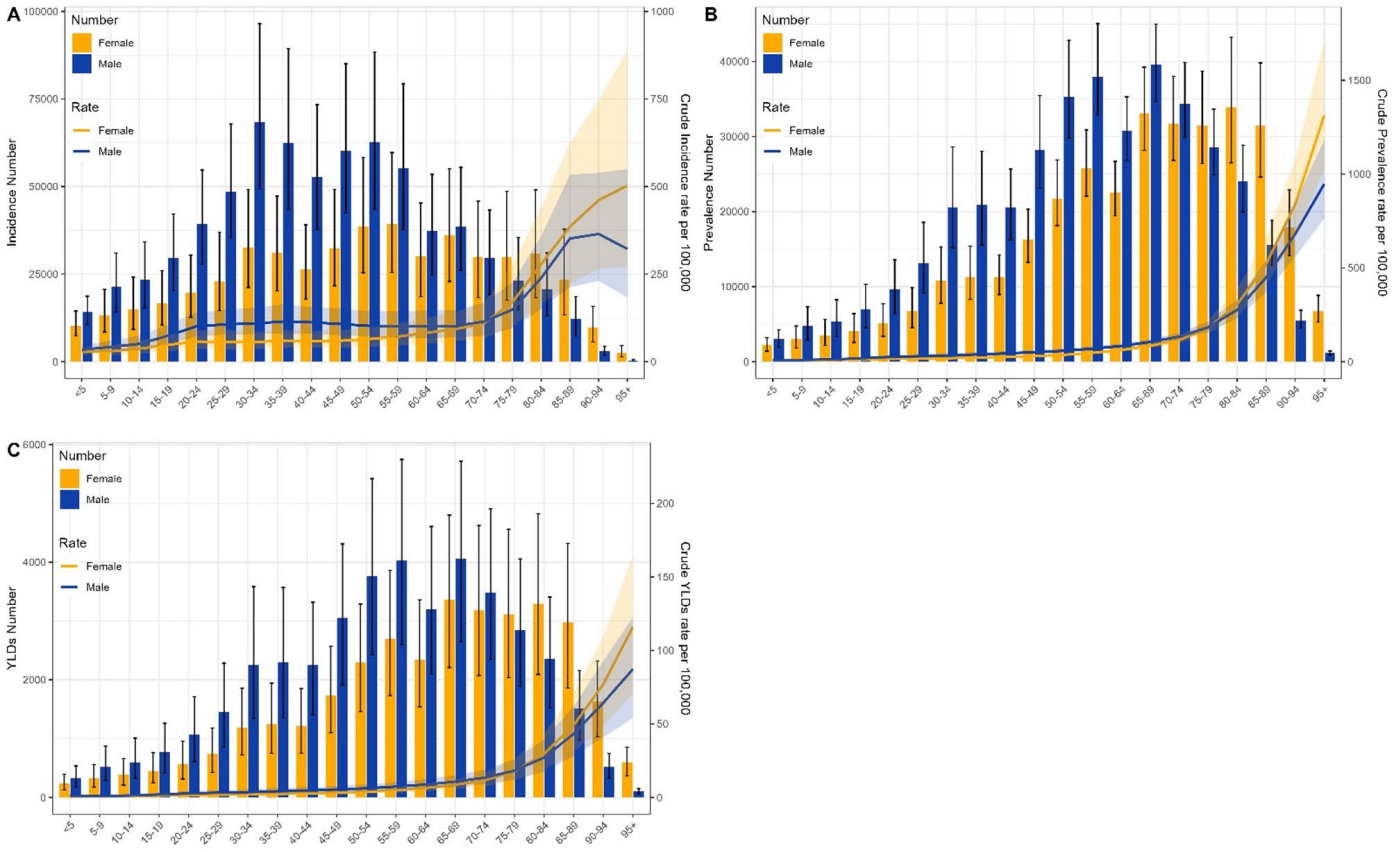
Burden of VFs in China by gender in 1990 and 2021. (**A**) Incidence number and CIR; (**B**) Prevalence number and CPR; (**C**) YLDs number and CYR

Globally, in 2021, the age group with the highest incidence of VFs was 30–34 for males and 65–69 for females. The age group with the highest prevalence was 70–74 for males and 80–84 for females. The age group with the highest YLDs was 65–69 for males and 80–84 for females. The age groups where females exceeded males in incidence, prevalence, and YLDs were 65–69, 70–74, and 70–74, respectively. Globally, the CIR, CPR, and CYR of VFs for both males and females increased with age, and females exceeded males in CIR, CPR, and CYR in the age groups of 65–69, 75–79, and 75–79, respectively (Supplementary Fig. 2). In China, in 2021, the age group with the highest incidence of VFs was 30–34 for males and 55–59 for females. The age group with the highest prevalence was 65–69 for males and 80–84 for females. The highest YLDs were in the age group of 65–69 for both males and females. The age groups where females exceeded males in incidence, prevalence, and YLDs were 70–74, 75–79, and 75–79, respectively. In China, the CIR, CPR, and CYR of VFs for both males and females increased with age, and females exceeded males in CIR, CPR, and CYR in the age groups of 75–79, 80–84, and 80–84, respectively (Fig. 4). It can be observed that, both globally and in China, the burden of VFs is heavier in males than in females in middle age, while in old age, the burden of VFs is heavier in females than in males.

### Decomposition analysis for the burden of VFs in China and globally

**Fig. 5:**
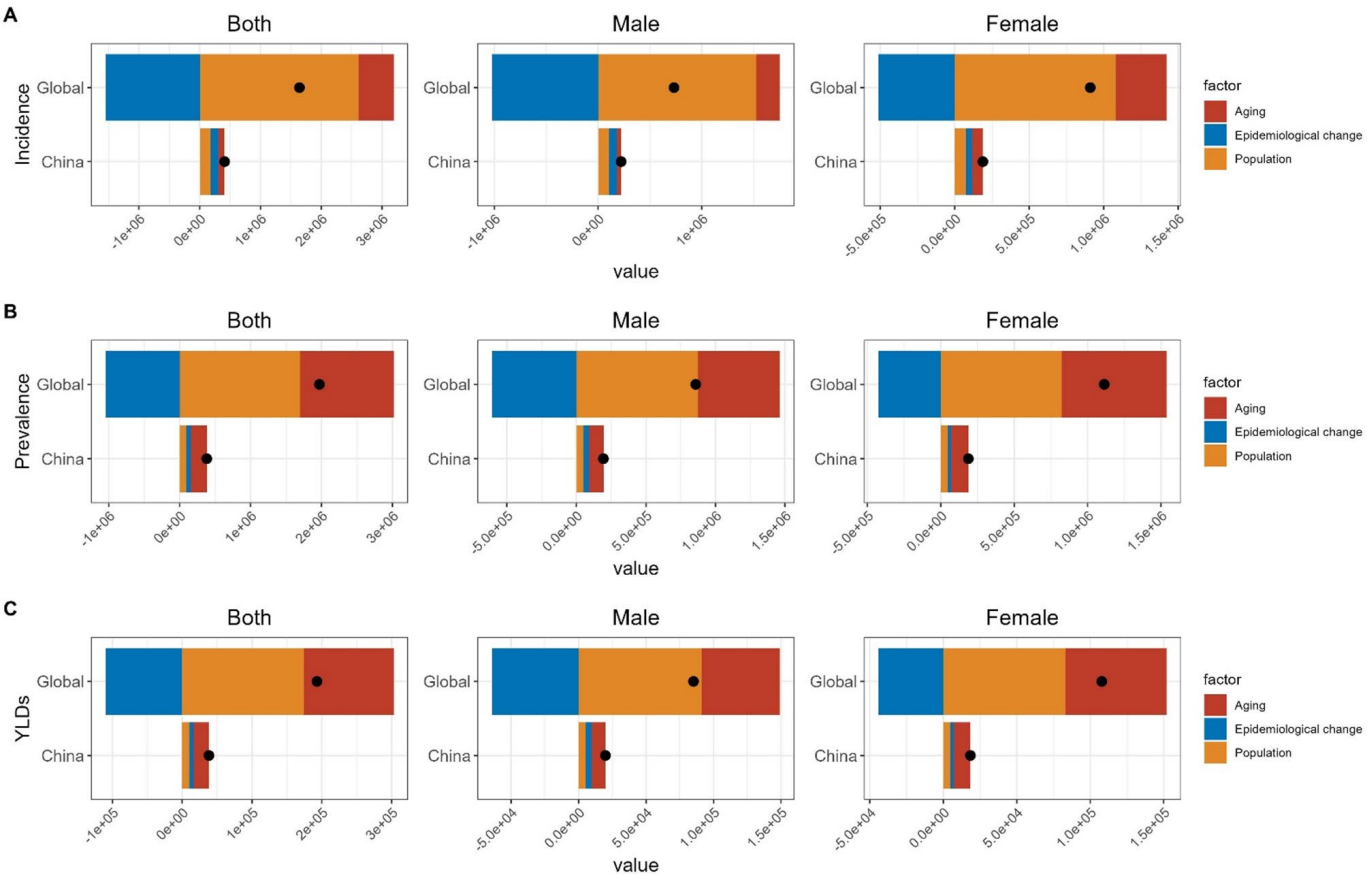
Decomposition analysis of the incidence, prevalence, and YLDs of VFs in China and globally from 1990 to 2021. (**A**) Incidence number; (**B**) Prevalence number; (**C**) YLDs number

From 1990 to 2021, the incidence, prevalence, and YLDs of VFs in China and globally increased significantly. This increase was decomposed into three aspects: aging, population growth, and epidemiological changes. Population growth was the main factor contributing to the increase in the incidence, prevalence, and YLDs of VFs globally. Its contribution in the overall population was 159.14%, 86.12%, and 90.3%, respectively. In the male population, it was 208.61%, 101.79%, and 106.89%, respectively. In the female population, it was 118.8%, 73.9%, and 77.03%, respectively. Epidemiological changes had a reducing effect globally. In China, population growth was the main factor contributing to the increase in the incidence of VFs. Its contribution in the overall, male, and female populations was 44.53%, 47.42%, and 40.38%, respectively. Aging was the main factor contributing to the increase in the prevalence and YLDs of VFs. Its contribution in the overall population was 58.39% and 57.53%, respectively. Among males, it was 53.49% and 52.47%, respectively. Among females, it was 63.1% and 62.66%, respectively. Epidemiological changes had an increasing effect in China (Fig. 5 and Supplementary Table 1).

## Discussion

This study used data from GBD 2021 to conduct an in-depth investigation into the epidemiological burden of VFs in China and globally from 1990 to 2021. The study results showed that the ASIR, ASPR, and ASYR indicators showed a continuous downward trend globally, but an upward trend in China. The prediction results indicated that these indicators will show a downward trend both globally and in China, but the decline in China is not as significant as that globally. The increase rate of VFs incidence, prevalence, and YLDs in China was twice the global level. Decomposition analysis further found that population growth and aging were the main factors contributing to the increase in the number of VFs cases in China. In terms of gender distribution, the burden of VFs in middle-aged men was greater than that in women, while the burden of VFs in elderly women was greater than that in men. The CIR, CPR, and CYR of VFs were positively correlated with patient age. Although China’s ASR-related indicators were lower than the global level, they showed an upward trend, and the increase rate of the corresponding number of cases was also higher than the global level. It is necessary to take tailored public health interventions for specific populations.

Compared with 1990, the incidence, prevalence, and YLDs of VFs in China and globally increased significantly in 2021, which may be related to population growth. The global population reached 7.8 billion in 2021, an increase of 47.2% compared with 1990 [17]. The latest national population census data in China in 2021 showed that its total population was 1.445 billion, an increase of 24.44% compared with 1990 [18]. In the decomposition analysis of this study, population growth was the main factor contributing to the increase in the incidence in China and globally, further supporting this conclusion. The ASIR, ASPR, and ASYR of global VFs showed a downward trend, which may not be due to the effective prevention of vertebral fractures, but due to the continuous increase in the global population. The global population increased by 47.2%, while the incidence only increased by 28.03%. Secondly, the term “global” used in the GBD study refers to the overall level of the global disease burden, rather than a specific region or country. In fact, some regions and countries show an upward trend, while others show a downward trend. It can be reasonably assumed that countries with a downward trend in VFs may offset the impact of countries with an upward trend, resulting in the overall downward trend globally from 1990 to 2021.

Unlike the global trend, China’s ASR-related indicators are on the rise, which may be associated with the increasingly severe aging of China’s population [19]. The elderly are prone to osteoporosis, and even minor trauma or daily activities can lead to fractures. According to statistics, in 2021, China’s population aged 60 and above was 264 million, accounting for 18.7% of the total population, and those aged 65 and above exceeded 190 million, accounting for 13.5% of the total population [18]. It is projected that China’s elderly population will grow rapidly in the future, with an average annual growth rate of 3.2%. The elderly population will exceed 300 million by 2026 and 400 million by 2041 [20]. By 2050, the population aged 65 and above will reach 400 million (26.9% of China’s population), and those aged 80 and above will be 150 million [21]. China’s aging population is becoming increasingly severe, and the decomposition analysis in this study confirmed that aging is the main factor contributing to the increase in the prevalence and YLDs of VFs. The analysis of age groups also supports the trend of the disease burden associated with aging. VFs pose a significant threat to the healthy living of the elderly and bring a huge economic burden to society [22]. Early identification of VFs in the elderly remains difficult because they may occur with low-trauma causes, are asymptomatic, and a large proportion of patients are incidentally diagnosed during radiological examinations of non-skeletal areas [23].

In terms of gender analysis, we found that the disease burden is heavier in elderly women than in elderly men. An epidemiological survey covering more than 95% of the urban population in China showed that from 2013 to 2017, among people aged 50 and above in China (average age 70.26 years), a total of 271,981 cases of vertebral fractures were found, of which 68.5% were women and 31.5% were men [9]. The heavier burden of VFs in elderly women is partly due to the long-term lack of estrogen after menopause, which leads to the overactivation of osteoclasts, enhances bone resorption, and thus causes bone loss [24]. On the other hand, women usually live longer than men, and the number of osteoporosis cases in elderly women is six times that of men [25], making them more likely to suffer from VFs. We also found that the disease burden of VFs is heavier in middle-aged men than in middle-aged women. This is because men often engage in high-risk jobs, such as construction workers, and are prone to falling from heights. Moreover, some behaviors of men, such as sports and violent injuries, can also cause VFs. Women, on the other hand, often engage in home-based activities such as childcare, which avoids some external risks [26, 27]. In addition, men are more likely to smoke and drink excessively than women, which can affect bone quality and increase their risk of fractures [28].

VFs are typical fractures of osteoporosis [29]. The incidence of osteoporotic fractures in China is steadily increasing [30], and the proportion of impaired self-care ability after vertebral fractures is as high as 23.8% [11]. Therefore, effective measures must be taken to address the problem of osteoporosis in China to reduce the risk of VFs. One study showed that exercise programs that include balance, functional, and resistance training are effective interventions to reduce falls in the elderly [31]. In addition, the implementation of the “5E” injury prevention strategy in some provinces of China has shown a decrease in the incidence of falls and the probability of subsequent falls [32]. Successful experiences can be promoted nationwide. Similarly, preventing refracture after VFs is also a serious challenge. In South Korea, the incidence of vertebral refracture four years after the first vertebral fracture is 27.53% [33]. In Japan, as many as 23.8% of patients do not receive systematic drug treatment after fracture [34]. Fracture Liaison Service (FLS) plays a crucial role in fracture management and has been proven to prevent subsequent fractures, reduce mortality, and be cost-effective [35]. In China, the concept and practice of FLS are being gradually promoted and applied. One experience from China showed that only 1 of the 226 patients followed up had a second fracture within 1 year [36]. It is expected that through raising public awareness, strengthening the training of health care professionals, optimizing resource allocation, and policy support, FLS will become more popular in China. This will help improve the quality of life of VFs patients.

At the same time, systematic osteoporosis treatment should be emphasized. A multicenter survey in China showed that among 1,993 fracture patients, only 69.6% received osteoporosis treatment after fracture, of whom 39.6% only received calcium supplements [11]. Bisphosphonates have played a role in the treatment of osteoporosis for decades. However, poor medication adherence, concerns about side effects, and the economic burden of long-term treatment have led to insufficient use of osteoporosis drugs, resulting in suboptimal disease treatment outcomes [37]. However, this can be managed through preventive strategies, enabling patients to benefit from anti-resorptive treatment [38]. New therapeutic drugs such as teriparatide and denosumab have shown higher efficacy in preventing fractures, but their widespread use is limited due to high costs, especially in low-income countries [39, 40]. To optimize the management of VFs, it is necessary to improve the knowledge level of healthcare providers, enhance patient consultation on medications and treatment plans, raise public awareness of osteoporosis risks, and implement supportive policies.

The aging situation in China is not optimistic, which will inevitably lead to an increase in the burden on families and the public health system [41]. To effectively address the challenges brought by the rapid aging of China’s population, it is necessary to take a multifaceted approach, considering the burden of aging and related fall risks. It is recommended to use channels such as television, radio, and social media to disseminate knowledge about the prevention and treatment of osteoporosis and related fractures. In addition, targeted public education activities should be carried out, such as health fairs and online education programs, to raise public awareness of osteoporosis and VFs. It is also recommended to provide continuing medical education courses for health care professionals to introduce the latest developments in the diagnosis and treatment of osteoporosis and its complications. Finally, it is recommended to encourage multidisciplinary team collaboration, including orthopedics, endocrinology, geriatrics, and rehabilitation, to provide comprehensive care for patients with VFs.

This study faces several limitations that should be acknowledged. First, the quality of GBD data varies across countries and regions. In some areas, such as rural and remote regions in China, the coverage of disease surveillance data is limited, which may lead to discrepancies between the estimated values and the actual situation. This quality disparity may have affected the accuracy of the DisMod MR model. It is recommended that future studies use various models for comparative analysis to reduce dependence on the output of a single model and improve the reliability of the estimates. Second, the use of medical imaging in clinical care has increased from 1990 to 2021, which may have led to an increase in the diagnosis of vertebral fractures in recent years compared with previous years, thus causing potential bias in the results.

## Conclusion

This study used the GBD 2021 database to comprehensively analyze the trends of VFs in China and globally by gender and age. VFs are a significant public health issue in China and globally, requiring adequate attention and corresponding resource allocation. In China, with the increase in population and the acceleration of population aging, VFs have become a key health issue for middle-aged and elderly people. Particularly for middle-aged men and elderly women. Therefore, developing health policies targeting VFs and intervening in the affected population in a timely manner may help to reduce the burden caused by VFs. It is suggested that future researchers should use these data critically and integrate evidence from other sources to develop more comprehensive and effective health strategies. In summary, the GBD 2021 database provides a powerful tool for understanding and addressing the burden of VFs in China and globally.

## Supplementary Information

Supplementary Material 1

## Data Availability

The data sets that were produced and/or evaluated within the scope of this study can be accessed through the Global Burden of Disease (GBD) database repository, which is located at http://ghdx.healthdata.org/gbd-results-tool.
