# Supplementary material for: Analysis of the disease burden of vertebral fractures in China and worldwide from 1990 to 2021 and trend forecast to 2035
Source: J Health Popul Nutr. 2025 Oct 28;44:378. doi: 10.1186/s41043-025-01114-8 (PMC12570628; doi:10.1186/s41043-025-01114-8)
Supplement: Supplementary file 1 — Supplementary Material 1 [file 41043_2025_1114_MOESM1_ESM.docx]

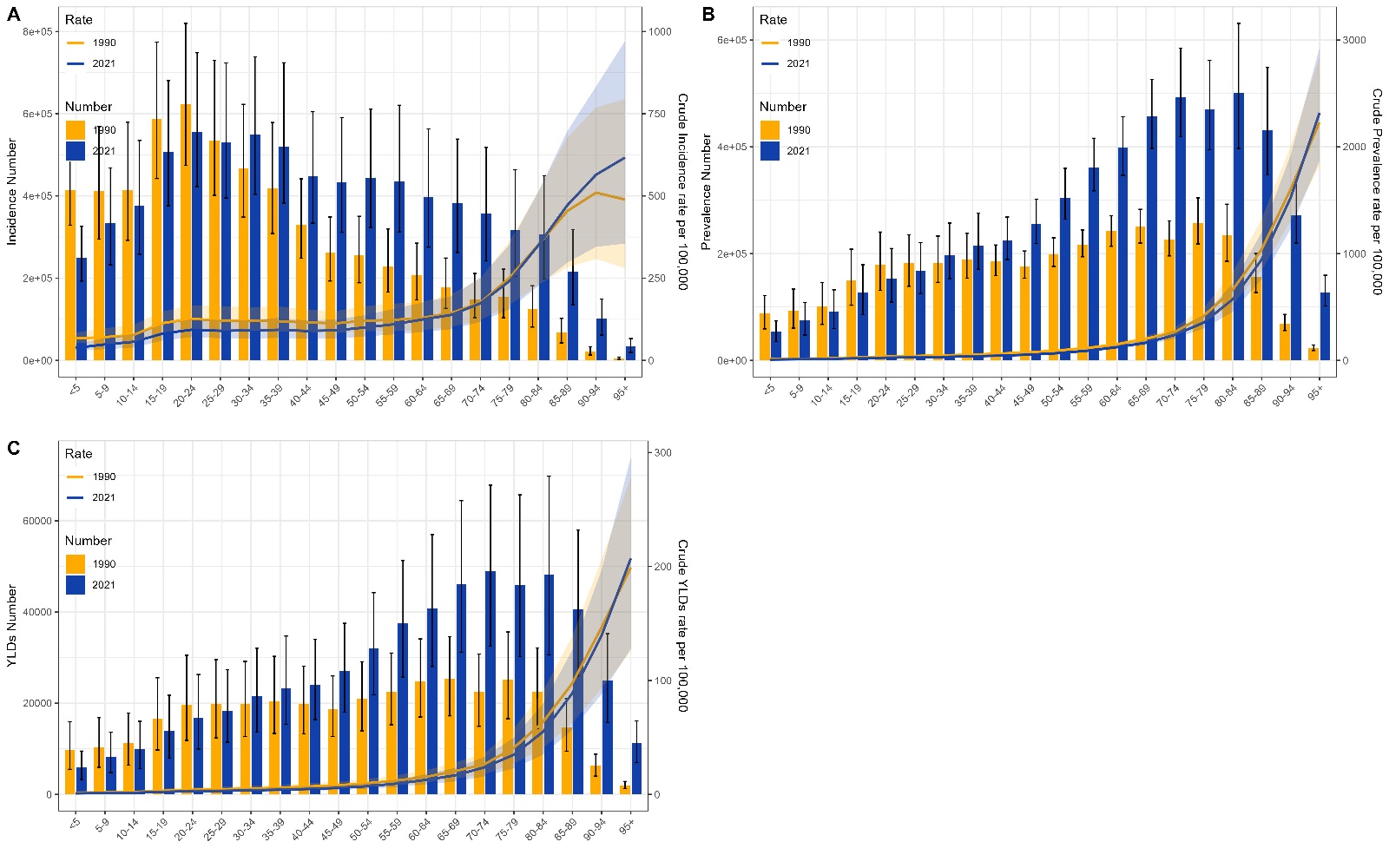


Supplementary Fig. 1 Burden of VFs in worldwide by age group in 1990 and 2021. (A) Incidence number and CIR; (B) Prevalence number and CPR; (C) YLDs number and CYR.


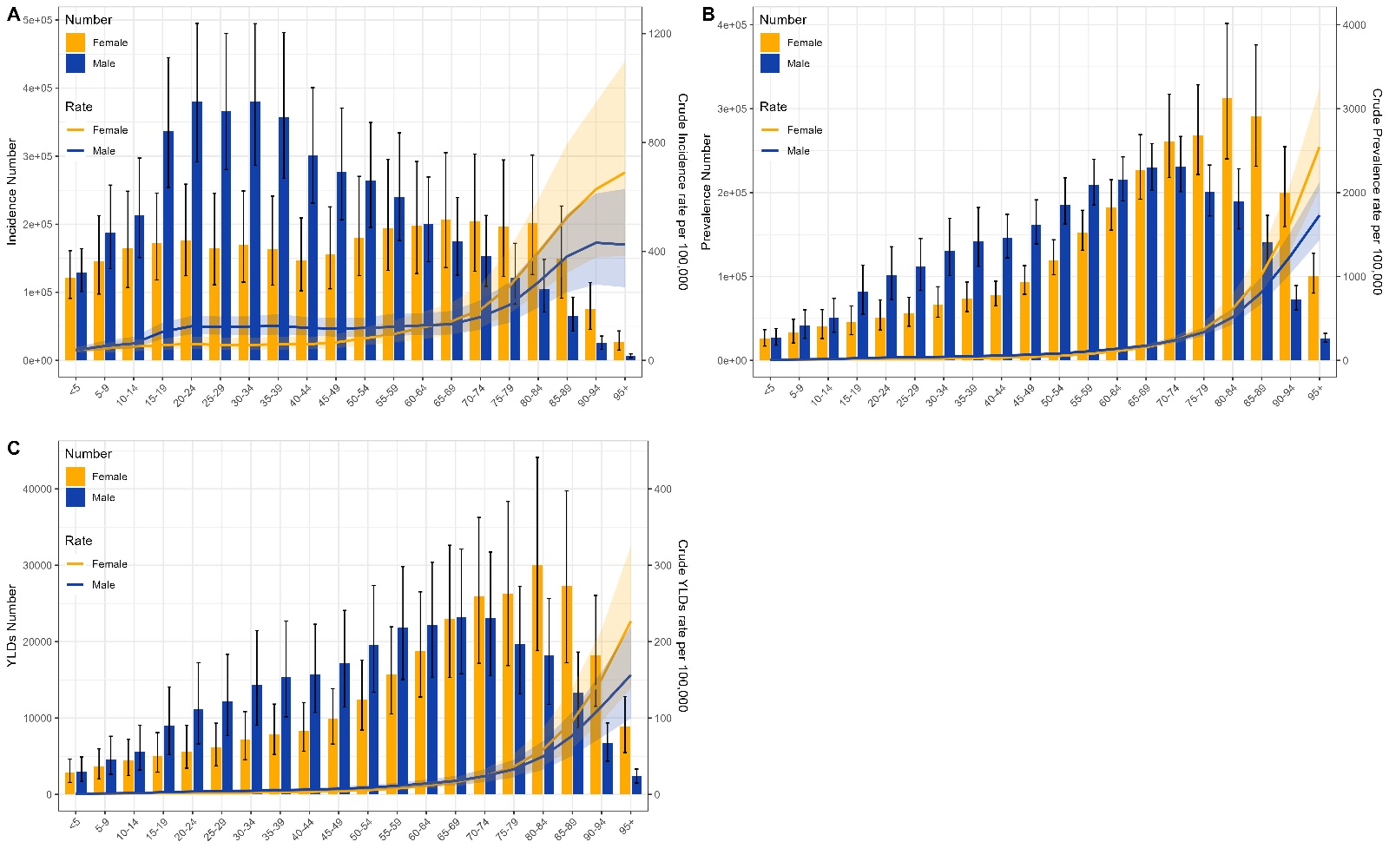


Supplementary Fig. 2 Burden of VFs in worldwide by gender in 1990 and 2021. (A) Incidence number and CIR; (B) Prevalence number and CPR; (C) YLDs number and CYR.

Supplementary Table. 1 Changes in Incidence, Prevalence and YLDs number according to population-level determinants from 1990 to 2021

| **Location** | **Measure** | **Sex** | **Overall difference** | **Change due to Population-level determinants**  **(% contribute to the total changes)** | | |
| --- | --- | --- | --- | --- | --- | --- |
|  |  |  |  | **Aging** | **Population** | **Epidemiological change** |
| China | Incidence | Both | 410079.64 | 110564.452(26.96) | 182611.295 (44.53) | 116903.888(28.51) |
|  |  | Male | 221562.26 | 41440.794 (18.7) | 105062.316 (47.42) | 75059.148 (33.88) |
|  |  | Female | 188517.38 | 70296.501 (37.29) | 76113.997 (40.38) | 42106.878 (22.34) |
|  | Prevalence | Both | 381463.97 | 222718.47 (58.39) | 95106.889 (24.93) | 63638.609 (16.68) |
|  |  | Male | 194313.93 | 103931.934(53.49) | 50230.231 (25.85) | 40151.765 (20.66) |
|  |  | Female | 187150.04 | 118097.033 (63.1) | 44527.312 (23.79) | 24525.695 (13.1) |
|  | YLDs | Both | 38328.93 | 22051.186 (57.53) | 9948.278 (25.96) | 6329.47 (16.51) |
|  |  | Male | 19884.71 | 10433.745 (52.47) | 5315.719 (26.73) | 4135.248 (20.8) |
|  |  | Female | 18444.22 | 11557.198(62.66) | 4590.933 (24.89) | 2296.092 (12.45) |
| Global | Incidence | Both | 1641219.83 | 579721.558(35.32) | 2611755.354(159.14) | -1550257.08(-94.46) |
|  |  | Male | 731287.69 | 228685.041(31.27) | 1525556.645(208.61) | -1022953.995(-139.88) |
|  |  | Female | 909932.14 | 341845.452(37.57) | 1080979.15 (118.8) | -512892.461 (-56.37) |
|  | Prevalence | Both | 1970978.02 | 1320386.541(66.99) | 1697430.152 (86.12) | -1046838.67 (-53.11) |
|  |  | Male | 857990.53 | 591328.704 (68.92) | 873391.123 (101.79) | -606729.297 (-70.72) |
|  |  | Female | 1112987.49 | 715411.792 (64.28) | 822467.309 (73.9 ) | -424891.608 (-38.18) |
|  | YLDs | Both | 192963.65 | 128561.051 (66.62) | 174248.536 (90.3) | -109845.936 (-56.93) |
|  |  | Male | 85137.17 | 58378.185 (68.57) | 91004.055 (106.89) | -64245.073 (-75.46) |
|  |  | Female | 107826.48 | 68894.902 (63.89) | 83063.585 (77.03) | -44132.003 (-40.93) |

**Guidelines for Accurate and Transparent Health Estimates Reporting (GATHER)**

This study adhered to the **GATHER guidelines** to ensure transparency, reproducibility, and reliability in reporting health estimates. The following outlines how the principles were applied in this research:

1. **Data Inputs**:

Data were sourced from the publicly available **Global Burden of Disease (GBD) 2021 repository**.

Inputs included estimates for incidence, prevalence, years lived with disability (YLDs), and disability-adjusted life years (DALYs) derived from vital records, population surveys, health service utilization data, disease registries, and verbal autopsy reports.

The GBD platform (<http://ghdx.healthdata.org/gbd-results-tool>) was used for data extraction.

1. **Model Specifications**:

The study employed the **DisMod-MR** meta-regression model, which synthesizes data from multiple sources to estimate disease burden while addressing data gaps and inconsistencies.

Covariates such as socio-economic status, healthcare access, and environmental risk factors were incorporated to account for missing or incomplete data.

1. **Estimation Methods**:

Joinpoint regression analysis was used to determine the annual percentage change (APC) in LBP metrics (ASIR, ASPR, and ASDR) and to identify significant shifts in trends over time.

Logarithmic transformations were applied to linearize exponential trends and improve model fit.

1. **Uncertainty Quantification**:

Uncertainty intervals (95% CI) were calculated for all estimates to reflect data variability.

Sensitivity analyses were performed to validate model robustness and account for variations in data quality across regions.

1. **Validation**:

Comparisons with prior GBD studies and independent sources were conducted to confirm the consistency of results.

Internal cross-validation of the DisMod-MR model was performed to ensure reliability.

1. **Limitations**:

The study acknowledges residual confounding due to regional disparities in healthcare infrastructure and reporting quality.

Data gaps in low-income and rural regions, particularly in China, may influence the precision of estimates despite imputation efforts.

1. **Accessibility**:

All data and estimation tools used in this study are publicly accessible through the **GBD repository** (<http://ghdx.healthdata.org/gbd-results-tool>), ensuring full reproducibility.
